# Supplementary figures and images for: A study on an efficient citrus Huanglong disease detection algorithm based on three-channel aggregated attention
Source: PeerJ Comput Sci. 2025 Jul 8;11:e2943. doi: 10.7717/peerj-cs.2943 (PMC12453638; doi:10.7717/peerj-cs.2943)

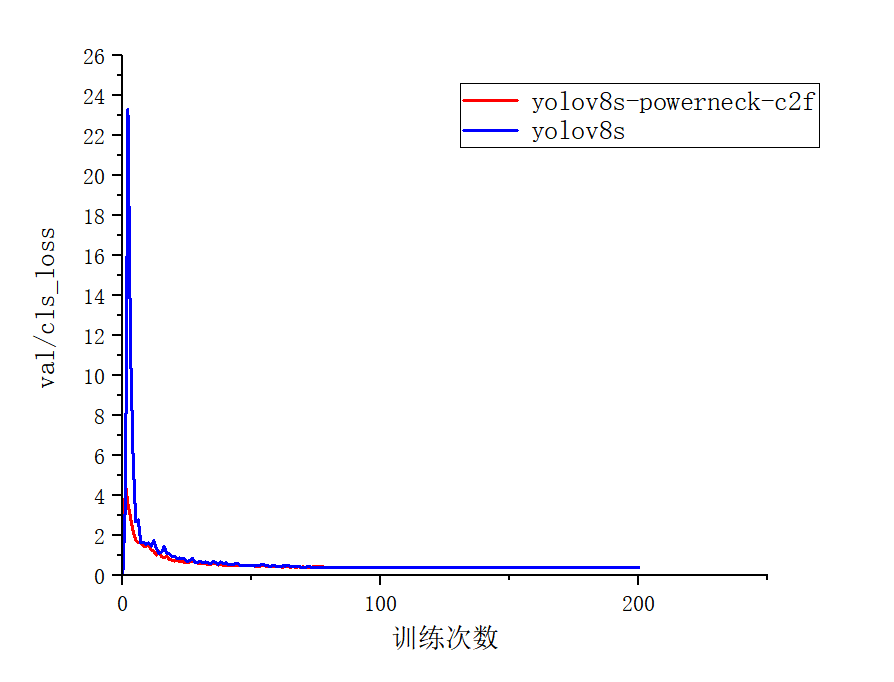

Supplement: Supplemental Information 2 [file peerj-cs-11-2943-s002.zip › Experimental Figures/Data Figures/08-Fig. 8 Plot of test bounding box loss function/Plot of test bounding box loss function.png]

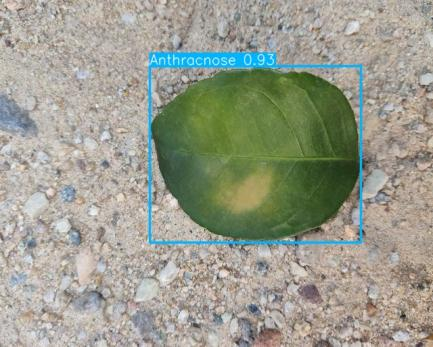

Supplement: Supplemental Information 2 [file peerj-cs-11-2943-s002.zip › Experimental Figures/Data Figures/11-Fig.11 YOLOv8 Improved Detection Result Chart/Anthracnose1.png]

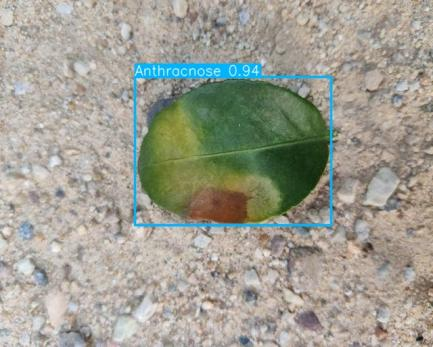

Supplement: Supplemental Information 2 [file peerj-cs-11-2943-s002.zip › Experimental Figures/Data Figures/11-Fig.11 YOLOv8 Improved Detection Result Chart/Anthracnose2.png]

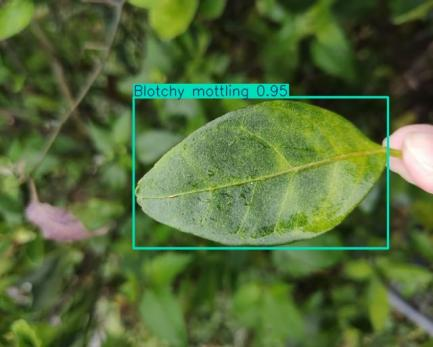

Supplement: Supplemental Information 2 [file peerj-cs-11-2943-s002.zip › Experimental Figures/Data Figures/11-Fig.11 YOLOv8 Improved Detection Result Chart/Blotchy motting1.png]

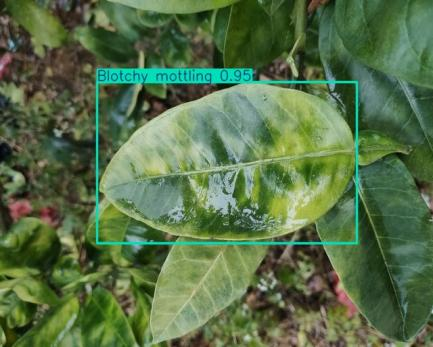

Supplement: Supplemental Information 2 [file peerj-cs-11-2943-s002.zip › Experimental Figures/Data Figures/11-Fig.11 YOLOv8 Improved Detection Result Chart/Blotchy motting2.png]

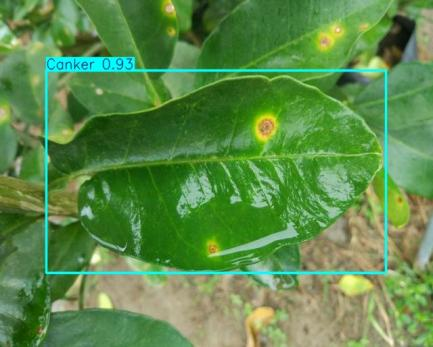

Supplement: Supplemental Information 2 [file peerj-cs-11-2943-s002.zip › Experimental Figures/Data Figures/11-Fig.11 YOLOv8 Improved Detection Result Chart/Canker1.png]

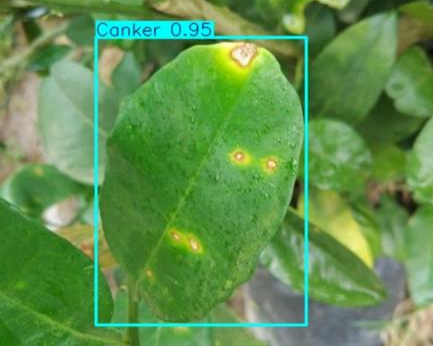

Supplement: Supplemental Information 2 [file peerj-cs-11-2943-s002.zip › Experimental Figures/Data Figures/11-Fig.11 YOLOv8 Improved Detection Result Chart/Canker2.png]

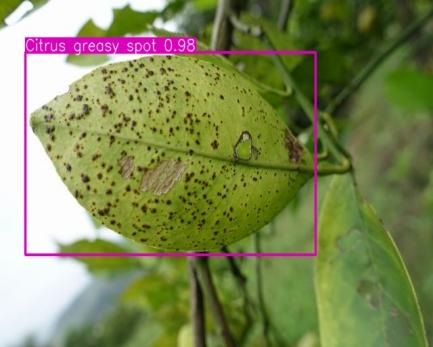

Supplement: Supplemental Information 2 [file peerj-cs-11-2943-s002.zip › Experimental Figures/Data Figures/11-Fig.11 YOLOv8 Improved Detection Result Chart/Citrus greasy spot1.png]

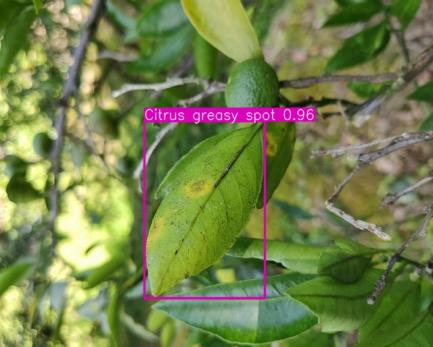

Supplement: Supplemental Information 2 [file peerj-cs-11-2943-s002.zip › Experimental Figures/Data Figures/11-Fig.11 YOLOv8 Improved Detection Result Chart/Citrus greasy spot2.png]

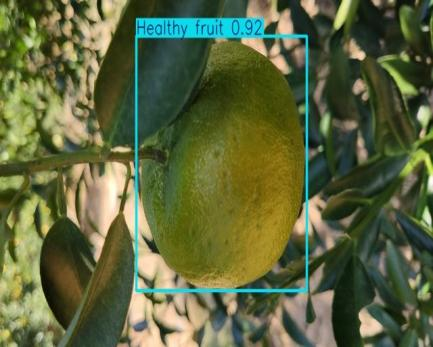

Supplement: Supplemental Information 2 [file peerj-cs-11-2943-s002.zip › Experimental Figures/Data Figures/11-Fig.11 YOLOv8 Improved Detection Result Chart/Healthy fruit1.png]

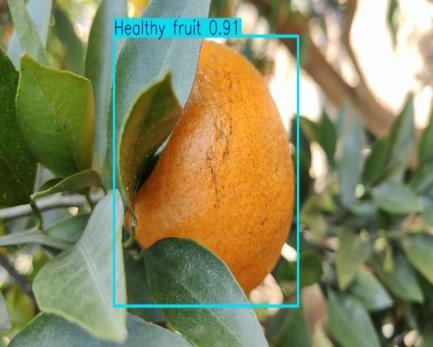

Supplement: Supplemental Information 2 [file peerj-cs-11-2943-s002.zip › Experimental Figures/Data Figures/11-Fig.11 YOLOv8 Improved Detection Result Chart/Healthy fruit2.png]

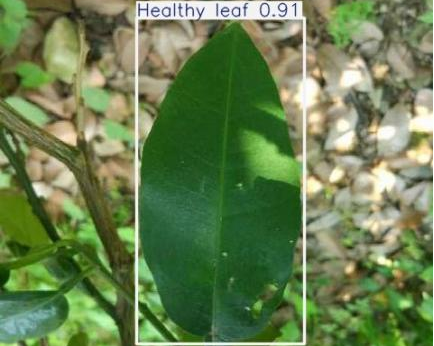

Supplement: Supplemental Information 2 [file peerj-cs-11-2943-s002.zip › Experimental Figures/Data Figures/11-Fig.11 YOLOv8 Improved Detection Result Chart/Healthy leaf1.png]

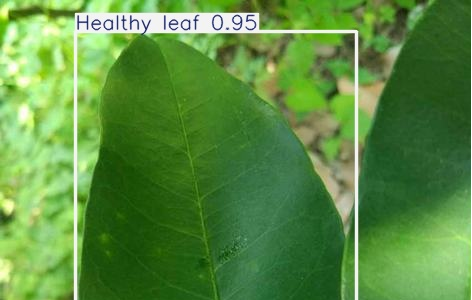

Supplement: Supplemental Information 2 [file peerj-cs-11-2943-s002.zip › Experimental Figures/Data Figures/11-Fig.11 YOLOv8 Improved Detection Result Chart/Healthy leaf2.png]

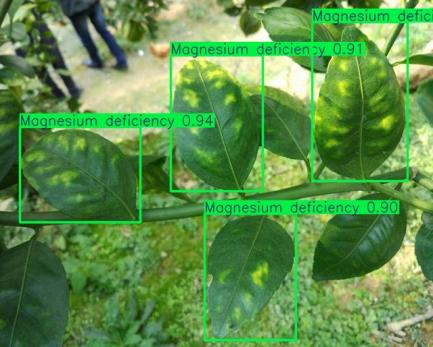

Supplement: Supplemental Information 2 [file peerj-cs-11-2943-s002.zip › Experimental Figures/Data Figures/11-Fig.11 YOLOv8 Improved Detection Result Chart/Maqnesium deficiency1.png]

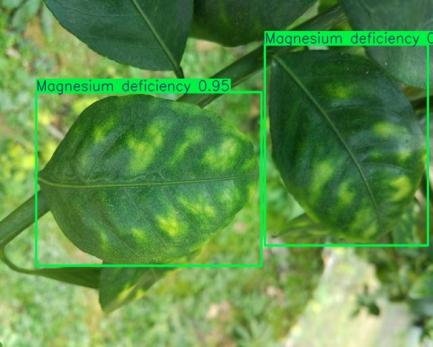

Supplement: Supplemental Information 2 [file peerj-cs-11-2943-s002.zip › Experimental Figures/Data Figures/11-Fig.11 YOLOv8 Improved Detection Result Chart/Maqnesium deficiency2.png]

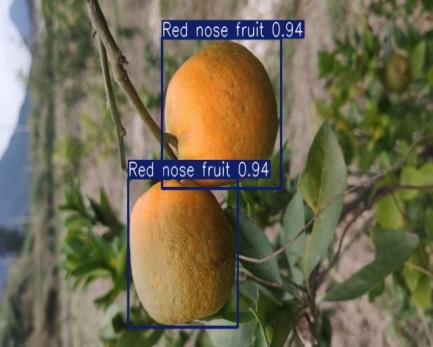

Supplement: Supplemental Information 2 [file peerj-cs-11-2943-s002.zip › Experimental Figures/Data Figures/11-Fig.11 YOLOv8 Improved Detection Result Chart/Red nose fruit1.png]

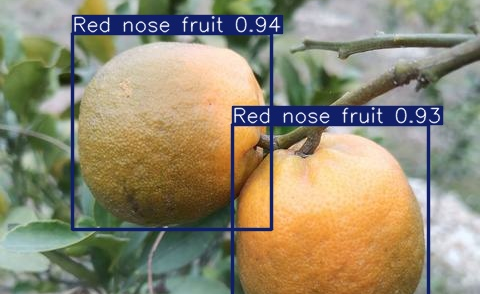

Supplement: Supplemental Information 2 [file peerj-cs-11-2943-s002.zip › Experimental Figures/Data Figures/11-Fig.11 YOLOv8 Improved Detection Result Chart/Red nose fruit2.png]

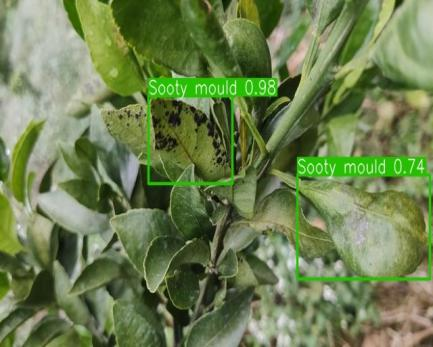

Supplement: Supplemental Information 2 [file peerj-cs-11-2943-s002.zip › Experimental Figures/Data Figures/11-Fig.11 YOLOv8 Improved Detection Result Chart/Soot mould1.png]

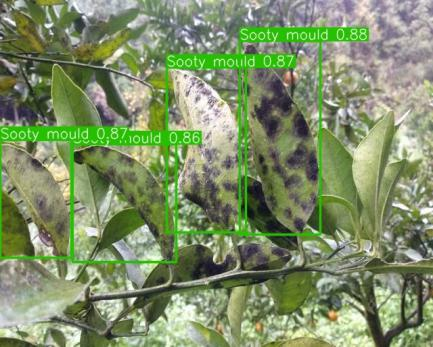

Supplement: Supplemental Information 2 [file peerj-cs-11-2943-s002.zip › Experimental Figures/Data Figures/11-Fig.11 YOLOv8 Improved Detection Result Chart/Soot mould2.png]

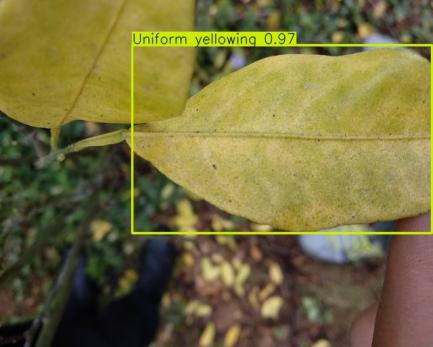

Supplement: Supplemental Information 2 [file peerj-cs-11-2943-s002.zip › Experimental Figures/Data Figures/11-Fig.11 YOLOv8 Improved Detection Result Chart/Uniform yellowing1.png]

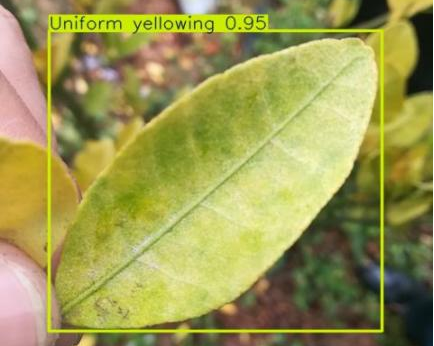

Supplement: Supplemental Information 2 [file peerj-cs-11-2943-s002.zip › Experimental Figures/Data Figures/11-Fig.11 YOLOv8 Improved Detection Result Chart/Uniform yellowing2.png]

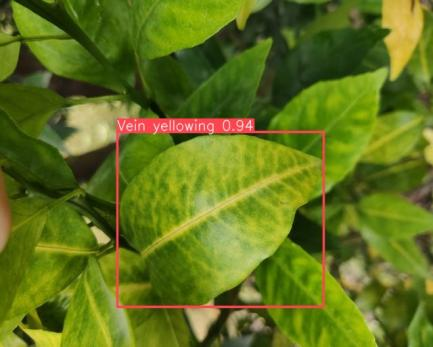

Supplement: Supplemental Information 2 [file peerj-cs-11-2943-s002.zip › Experimental Figures/Data Figures/11-Fig.11 YOLOv8 Improved Detection Result Chart/Vein yellowing1.png]

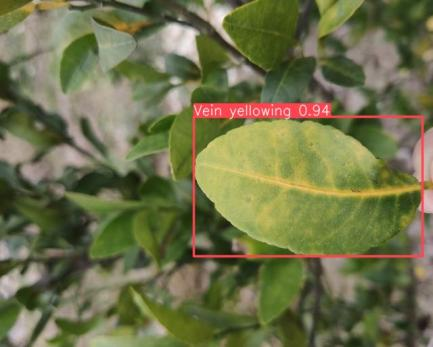

Supplement: Supplemental Information 2 [file peerj-cs-11-2943-s002.zip › Experimental Figures/Data Figures/11-Fig.11 YOLOv8 Improved Detection Result Chart/Vein yellowing2.png]

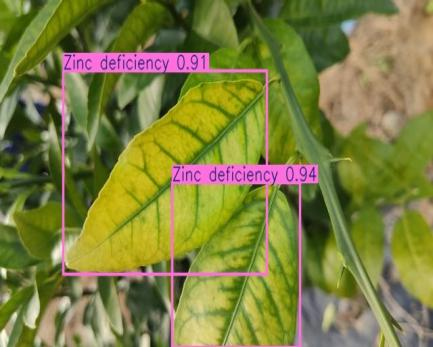

Supplement: Supplemental Information 2 [file peerj-cs-11-2943-s002.zip › Experimental Figures/Data Figures/11-Fig.11 YOLOv8 Improved Detection Result Chart/Zinc deficiency1.png]

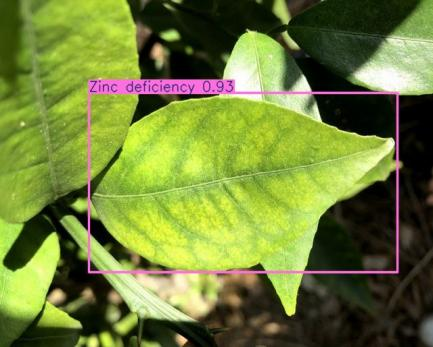

Supplement: Supplemental Information 2 [file peerj-cs-11-2943-s002.zip › Experimental Figures/Data Figures/11-Fig.11 YOLOv8 Improved Detection Result Chart/Zinc deficiency2.png]
